# Impact on the Mental and Physical Health of the Portuguese Population during the COVID-19 Confinement

**DOI:** 10.3390/jcm10194464

**Published:** 2021-09-28

**Authors:** Fátima Frade, Lia Jacobsohn, Juan Gómez-Salgado, Rosário Martins, Regina Allande-Cussó, Carlos Ruiz-Frutos, João Frade

**Affiliations:** 1 Escola Superior de Enfermagem de Lisboa, 1600-190 Lisboa, Portugal; fatimafrade4@sapo.pt; 2 Centro de Administração e Políticas Públicas (CAPP), University of Lisbon, 1300-666 Lisbon, Portugal; 3 Escola Superior de Saúde, Universidade Atlântica, 2730-036 Barcarena, Portugal; ljacobsohn@uatlantica.pt; 4 Centro de Medicina de Reabilitação do Alcoitão, 2649-506 Alcabideche, Portugal; 5 Department of Sociology, Social Work and Public Health, Faculty of Labour Sciences, University of Huelva, 21007 Huelva, Spain; frutos@uhu.es; 6 Safety and Health Postgraduate Programme, Universidad Espíritu Santo, Guayaquil 092301, Ecuador; 7 Escola Superior de Saúde, Instituto Politécnico de Viana do Castelo, 4900-347 Viana do Castelo, Portugal; martins.maria@ess.ipvc.pt; 8 Nursing Department, University of Seville, 41009 Sevilla, Spain; rallande@us.es; 9 Instituto Politécnico de Leiria, Escola Superior de Saúde e Centre for Innovative Care and Health Technology (ciTechcare), 2411-901 Leiria, Portugal; joao.frade@ipleiria.pt; 10 Unit for Multidisciplinary Research in Biomedicine (UMIB), University of Porto, 4099-002 Porto, Portugal

**Keywords:** COVID-19, psychological distress, pandemic, public health, quarantine, mental health

## Abstract

Confinement of the population has been one of the measures implemented by different governments to address the COVID-19 health crisis, and it has led to social isolation together with a disruption of daily activities. The aim of the study is to analyze psychological distress during the COVID-19 pandemic in Portugal. During the quarantine, a cross-sectional study was carried out on a sample of 2120 subjects over 18 years of age, resident and born in Portugal. Data were collected using a self-developed questionnaire that considered socio-demographic variables, physical symptoms, health conditions, and history of contact with COVID-19, as well as psychological alterations. The General Health Questionnaire (GHQ-12) was also included. Univariate and bivariate statistical analyses were performed. Predictive capacity was studied using logistic regression models. The results showed a higher percentage of individuals presenting psychological distress (57.2.0%), with a higher percentage identified among women (79.0%), and in people with a higher educational level (bachelor’s + master’s and doctorate) (75.8%). The predictor variables with the greatest weight were sex, educational level (graduation, master’s, and doctorate), living with children or under 16 years of age, presence of symptoms, and quarantine in the last 14 days for having symptoms. Good self-assessment of health and working at home appear to be protective against psychological distress. These results highlight the impact of the COVID-19 pandemic on psychological distress and provide an opportunity to consider the need to implement specific multidisciplinary public health and mental health interventions in this pandemic situation.

## 1. Introduction

In 2019, the world was surprised by a pandemic caused by the new coronavirus (SARS-CoV-2), which was called COVID-19. COVID-19 began in Wuhan, China, and soon spread to the rest of the world. As of February 2021, there were 111,102,016 cases of infection by the new coronavirus and 2,462,911 deaths worldwide [1]. In Portugal, the number of confirmed cases of COVID-19 infection is 798,074 and the number of deaths is 16,023 [2]. Being an unknown virus, much research has been done in relation to it and also in relation to the impact it has had on the physical and psychological health of people living through the pandemic caused by it. The main physical signs and symptoms of the infection are fever, dry cough, dyspnea, odynophagia, headache, myalgia, chills, nausea, vomiting, diarrhoea, hemoptysis, and conjunctival congestion. The main psychological effects of the virus influence people’s mental health, causing higher levels of stress, anxiety, psychological distress, and depression [3,4,5,6,7,8].

In the specific case of Portugal, in response to the rapid epidemiological evolution of the COVID-19 disease, and with the aim of containing the virus and moderating the social, health, and economic impact of the pandemic, a state of alarm was declared on 18 March 2020 [9]. During the state of emergency, mandatory confinement measures were adopted for infection control, like the limitation of free movement of citizens, favoring teleworking (except for essential professions for basic needs like health, food, and safety), face-to-face teaching activities were suspended in favor of online teaching (in different levels of education), and cultural, sporting, and religious activities were suspended. The obligation to maintain social distance, use of masks, and compliance with respiratory etiquette was maintained. Deconfinement began at the beginning of May. At this stage, the general infection control measures included the mandatory use of a mask; compliance with respiratory etiquette; maintaining social distance; avoiding contact with people who present symptoms suggestive of COVID-19; working from home whenever possible (telework); prioritizing using the telephone or electronic services to get in touch with other services, such as supermarkets, pharmacies, or other; contacting the health services in advance in case of need for medical care; and avoiding crowded places, unnecessary contacts (inside or outside the home), and promotion of or participation in events that bring together many people [2,9]. 

The impact of the COVID-19 pandemic on the physical and mental health of the population is undeniable, and the published evidence describes it as such [10]. Thus, it is estimated that 38.2% of the European population has psychological disorders related to COVID-19 [11]. Social distancing and self-isolation during the COVID-19 pandemic have challenged people’s mental health and general well-being, contributing to increased mental health problems which include depression, anxiety, mood disorder, psychological distress, post-traumatic stress disorder, insomnia, fear, stigma, lack of self-esteem, and lack of self-control [12].

The risk factors that compromise the psychological well-being of people experiencing the COVID-19 pandemic are fear of not having economic conditions for the goods of first necessity and for food, fear of quarantine, level of health perception, degree of risk control, and risk perception. Other studies reveal, as risk factors for psychological well-being, the perception of increased risk of SARS, a history of contact with people tested positive, feeling symptoms similar to those of SARS, loss of social contact and breaking of family routines, and increase of sedentary behaviors [7,12,13,14,15,16]. Given the variability in the risk factors for developing psychological distress, and the protective factors identified, it is worth considering the need for further studies to refine these results. Despite the agreement on the presence of psychological distress during the COVID-19 pandemic situation, the characteristics of the population are disparate, and more variables need to be controlled [13]. 

In Portugal, the few studies conducted during the COVID-19 pandemic show that the Portuguese population deteriorated in their mental health conditions during the pandemic, and the percentage of anxiety and depression is evident [14]. Other symptoms presented by the Portuguese population are poor sleep quality, insomnia, fear, anxiety, depression, and obsessive-compulsive symptoms due to COVID-19 [15,16]. 

Studies carried out in Portugal have focused on identifying the percentages of psychological factors in the Portuguese population during the pandemic and listing different symptoms. In our study, in addition to identifying the psychological factors present in the Portuguese population, we also identify the factors that predict their development, thus making it possible to plan action measures that can be implemented with the aim of reducing the presence of these factors in other pandemic situations. Thus, the aim of this study is to analyze psychological distress in a Portuguese population sample during the COVID-19 pandemic to identify the existence of related sociodemographic and specific health factors.

## 2. Materials and Methods

### 2.1. Design

The study design was cross-sectional and observational.

### 2.2. Sample

The initial sample consisted of 3105 participants. Of these, 985 were discarded because they did not complete the questionnaire in its entirety. Thus, the final sample was 2120 people, recruited between April and October 2020.

The inclusion criteria for participants were: (i) 18 years old or older; (ii) Portuguese residents during the COVID-19 pandemic, and (iii) having accepted the informed consent.

### 2.3. Variables 

Regarding socio-demographic variables, sex, age, marital status, level of education, employment status, number of people living with, living with a child or adolescent, and living with a disabled person were considered. Likewise, with regard to the specific situation related to COVID-19, the presence of symptoms, self-perceived level of health, and history of contact and/or exposure were also identified. Considering these variables as independent variables, psychological distress was set as a dependent variable.

It is worth mentioning that whether the items related to COVID-19 symptoms were present in the last 14 days was asked about, and the response options were listed as those stipulated by the World Health Organization. These included fever of 38 °C or higher, cough, headache, myalgia, dizziness, diarrhoea, sore throat, coryza, chills, and shortness of breath.

Following Wang et al. [17], physical and mental health status were evaluated by dichotomous (“Yes/No”) questions, as follows: having a chronic illness, having a disability, being on medication, having been hospitalised in the last 14 days, and having attended a health service in the last 14 days.

Other variables were contact history and health status, using the Ilder and Benyamini approach, with small variants, in the current pandemic situation [17,18,19,20,21].

### 2.4. Instruments

An ad hoc questionnaire was designed that included all the variables described. Psychological adjustment was assessed using the General Health Questionnaire (GHQ-12), duly validated for the Portuguese population [22] and used as a screening tool for non-psychotic mental disorders in other pandemic situations [22,23]. This questionnaire consists of 12 items with four response options. If the participant chooses options 1 or 2, then the final score for this item is 0 points; however, if the participant selects options 3 and 4, the score of this item is 1 point. After calculating the total sum of these scores, the final range is between 0 and 12 points. 

For the present study, the overall score with reliability estimated by Cronbach’s alpha at 0.851 was used as a single factor. The cut-off point for the general population was 3, where subjects with scores greater than or equal to 3 were considered more likely to develop psychiatric morbidity [24,25].

### 2.5. Procedures

Initially, a rapid literature review was conducted regarding the psychological effects that other pandemics, and the protective measures implemented, had caused in the population. Based on these results, a preliminary version of the questionnaire was designed and assessed by a panel of experts composed of 10 professionals (three doctors, four nurses, and three psychologists, two of them specialists in clinical psychology). Once the tool had been refined and the relevant modifications had been made, a pilot study was carried out on a sample of 57 subjects, identified by non-probabilistic sampling, over 18 years of age, seeking a similar proportion between men and women (50.9% and 49.1%, respectively). The mean age of this sample of subjects was 41.87 years (SD = 11.86), 56.1% reported being married, and 57.9% reported having completed a master’s or doctoral degree.

No subjects in the pilot study reported problems in understanding the items, and no problems were detected in the design and visualization of the survey on different devices (PC, tablet, or smartphone). 

Qualtrics^®^ XM platform was the software used for the creation, design, and dissemination of the survey in this study. For the field study, a non-probabilistic snowball sampling was also implemented, based on the dissemination of the link via email lists to universities and professional associations, who were asked to help in this dissemination process.

### 2.6. Ethical Considerations

The ethical principles set out in the Declaration of Helsinki [26] have been followed. Participants’ permission was obtained by means of an informed consent form where they expressed their voluntary desire to participate. Data were recorded in an anonymous way and treated confidentially. The project was approved by the Research Ethics Committee of Atlântica—Instituto Universitário.

### 2.7. Data Analyses

A descriptive univariate and bivariate statistical analysis was carried out, after studying the normality of the data distribution, using SPSS (26.0) software (IBM, Armonk, NY, USA). Measures of central tendency and dispersion were used for quantitative variables, and frequencies and percentages for qualitative variables. For the bivariate analysis, Chi-squared and Student’s *t*-statistics were used. Crammer’s V and Cohen’s d effect size indices were also calculated, considering the following cut-off points: 0 to 0.19, insignificant; 0.20 to 0.49, small; 0.50 to 0.79, medium; 0.80 and above, high.

After this, a logistic regression algorithm was run, controlling for sex and age, and including in the models tested the variables that proved to be significant (*p* < 0.05). Finally, a global predictive model was designed, which corresponds to model 5, where the relationship of all variables with the presence or absence of psychological problems is analyzed, that is, the predictive factors that predispose a person to the existence of psychological problems are analyzed by calculating the odds ratios (OR) with a 95% confidence interval.

## 3. Results

### 3.1. Psychological Distress

Table 1 shows the mean scores and standard deviations of the answers to the General Health Questionnaire questions given by the subjects.

The mean score for the total of the 12 points scale was 3.97 (SD = 3.43). Establishing a cut-off point of 3 or more points, 57.2% of the 2120 study participants presented psychological distress. Item 5, “Have you felt constantly overwhelmed and stressed?” (M = 2.68; SD = 0.90), and item 7, “Have you been able to enjoy your normal daily activities?” (M = 2.62; SD = 0.89), were the ones with the highest scores. In opposition, item 11, “Have you thought that you are a worthless person?” (M = 1.44; SD = 0.78), and item 10, “Have you lost confidence in yourself?” (M = 1.69; SD = 0.85), presented lower scores.

### 3.2. Sociodemographic Variables and Psychological Distress

With regard to the sociodemographic variables (Table 2), statistically significant differences were found between both groups as regards sex (χ^2^ = 163.137, *p* < 0.001, V = 0.277). No statistically significant differences were observed in the presence of psychological distress with age (χ^2^ = 1.394, *p* = 0.163, V = 0.063).

Of the total number of subjects who presented psychological distress (1212 subjects, 57% of the sample), 79% were women, 54.8% were married or cohabiting, 75.8% had completed university studies (university studies: graduation, master’s, and doctorate), and 69.2% worked outside the home. Regarding the conditions of the usual dwelling, 59.2% lived in a flat, and 50.2% lived with children under 16 years of age.

Statistically significant differences were also found regarding the variables marital status (χ^2^ = 13.353, *p* = 0.001, V = 0.079), employment status (χ^2^ = 8.328, *p* = 0.016, V = 0.063), and type of dwelling (χ^2^ = 5.011, *p* = 0.025, V = 0.063). 

The percentage of high education (graduation + master’s and PhD) status among those with psychological distress was 75.8%. In addition, as regards participants living with children or youngsters under the age of 16 (50.2%), a low percentage of psychological distress was described.

### 3.3. Physical Symptoms in the Past 14 Days and Psychological Distress 

Regarding the presence of symptoms in the 14 days prior to participation in the study, the following was distribution was obtained (Table 3): headache (46.6%), coryza (30.1%), myalgia (24.7%), cough (15.3%), sore throat (14.7%), and to a lesser extent, subjects reported suffering from diarrhea (9.7%), dizziness (9.7%), chills (5.2%), dyspnea (3.6%), and fever above 38 °C for at least one day (1.1%).

For all variables described in Table 3, statistically significant differences were found between the presence of physical symptoms and psychological distress (*p* < 0.005), excluding respiratory distress (*p* = 0.082) and fever (>38 °C for at least 1 day) (*p* = 0.319).

Statistically significant differences were also observed for the mean number of symptoms (χ^2^ = −13.150, *p* < 0.001, Cohen’s d = 0.559), with a mean effect size. The group of subjects with psychological distress had a higher number of symptoms (M = 1.58, SD = 1.55) compared to the group without this psychological morbidity (M = 0.80, SD = 1.16).

### 3.4. Health-Related Variables and Psychological Distress

Regarding health-related variables, the results show (Table 4) that 29% of the respondents reported having a chronic disease, and that 35.8% of the respondents are currently taking medication. In the same way, 0.4% of the subjects referred to having been hospitalized in the last 14 days, and 5.3% referred to having received health care in a health center, clinic, or hospital, while 4.5% of the participants had symptoms of COVID-19 and 18.1% reported diagnostic testing.

All the variables described in Table 4 are related to the presence of psychological distress, excluding recent hospitalization in the last 14 days (χ^2^ = 1.568, *p* = 0.211, Cohen’s d = 0.027).

Considering the subjects’ assessment of their self-perceived health in the last 14 days, statistically significant differences were also found between the two groups (t = 11.930, *p* < 0.001, Cohen’s d = 0.520), with a mean effect size. The group of subjects without psychological distress (M = 4.26, SD = 0.73) expressed a better self-perception of their health compared to the group with psychological distress (M = 3.85, SD = 0.83), although both groups have good self-rated health in the last 14 days (M = 4.02, SD = 0.81).

### 3.5. Variables Related to Contact History in the Past 14 Days and Psychological Distress

As regards contact history in the last 14 days (Table 5), 36.1% of the participants reported having had or not knowing if they had had close contact with an individual with confirmed infection with COVID-19. Of these, 37.1% of respondents claimed that the contact was casual, and 43.0% stated they had had or did not know if they had had contact with any person or material suspected of being infected with COVID-19. As for the presence of people infected with COVID-19 in the participants’ immediate circle, 91.2% indicated not having any infected family member. A statistically significant relationship with the presence of psychological distress (*p* ≤ 0.001 in all cases) was found for all contact history variables in the last 14 days. Nevertheless, the effect sizes were negligible.

### 3.6. Prediction of Psychological Distress 

The results of the logistic regression analysis controlled for sex and age are presented in Table 6. This model shows a variance of 22.6% in the overall model, with percentages of correct classification of each model around 68.3%. The model had a good fit (Hosmer–Lemeshow Chi-square value = 4.929, *p* = 0.765) and made it possible to identify the predictor variables of psychological distress.

Model 1 (sociodemographic variables) indicated a predictive ability of 12.7% (χ^2^ = 211.457, *p* < 0.001). Gender, specifically female (OR = 3.142, 95% CI = (2.566, 3.848)), educational level, employment status, and living with children or under 16 years of age were predictors. This model correctly classified 65% of the subjects with sensitivity and specificity parameters of 79.3% and 45.8%, respectively. 

With model 2, relating to physical symptoms, the value of the variance explained amounted to 16% (χ^2^ = 268.399, *p* < 0.001). 

Participants who reported a greater number of symptoms in the 14 days prior to study participation (OR = 1.444, 95% CI = (1.339, 1.558)) were more likely to show psychological distress. This model correctly classified 65.4% of participants (sensitivity 81.3% and specificity 44.3%).

Model 3 showed a predictive capacity of 16.8% (χ^2^ = 281.293, *p* < 0.001), slightly higher than the previous one, and included health-related variables. This model provided sensitivity and specificity values of 83.8% and 42.8%, correctly classifying 66.3% of the sample. Participants who scored higher on self-perceived health (OR = 0.563, 95% CI = 0.496, 0.640) were less likely to experience psychological distress. However, subjects who had been quarantined in the past 14 days for having symptoms were 2.878 times more likely to have psychological distress (95% CI = 1.456, 5.687).

Model 4 included contact history variables, which provided an explained variance rate of 13.1% (χ^2^ = 217.047, *p* < 0.001). Having close contact with an individual with confirmed COVID-19 infection (OR = 1.499, 95% CI = 1.120, 2.007), as well as having had any contact with any person or material suspected of being infected (OR = 1.437, 95% CI = 1.108, 1.863) had predictive ability, correctly classifying 65.3% of participants (80% sensitivity and 45.6% specificity).

Finally, Model 5 (global model), which contained the variables with predictive capacity in the previous models, showed a predictive capacity of 22.6%, correctly classifying 68.5% of the participants (78% sensitivity and 54.9% specificity). The variables that showed greater weight, with ORs greater than 1, were sex (OR = 2.448, 95% CI = 1.980, 3.028), educational level (OR = 1.419, 95% CI = 1.170, 1.820), living with children or children under 16 years of age (OR = 1.580, 95% CI = 1.304, 1.915), number of symptoms presented in the last 14 days (OR = 1.327, 95% CI = 1.224, 1.440), having been quarantined in the past 14 days for presenting symptoms (OR = 2.443, 95% CI = 1214, 4.913), having had close contact with an individual with confirmed COVID-19 infection (OR = 1.347, 95% CI = 1.031, 1.759), and having had contact with any person or material suspected of being infected with COVID-19 (OR = 1.237, 95% CI = 0.958, 1.598). Other predictor variables with ORs less than 1 were self-perceived health in the last 14 days and employment status.

## 4. Discussion

In this study conducted in Portugal, 57% of the participants present psychological distress during the COVID-19 pandemic, revealing that they often feel oppressed and tense and that they cannot enjoy the activities they usually perform in their daily life (i.e., here is where the suffering is greatest). Other studies corroborate our results, showing the presence of psychological distress in people during the COVID-19 pandemic. In Spain, a study revealed that a high percentage (i.e., 72%) of participants were at risk of developing psychological problems [7]. In China, a study found that 22.8% of participants reported high levels of psychological distress [27]. In the United States, the percentage of individuals reporting psychological distress was 73% in a study conducted at the beginning of the COVID-19 pandemic [28]. In Italy, the psychological impact that the COVID-19 pandemic caused was around 48.6% [12]. Studies that focused their attention on the psychological impact during the pandemic reveal that the percentage of psychological distress is between 22.9% and 56.7% [20,29,30]. The percentage of psychological distress in our study and other studies reported here exceeds the pandemic data, so we can interpret this to mean that the COVID-19 pandemic has affected populations more severely than other previous pandemics.

Regarding the sociodemographic variables for which there is greater vulnerability to psychological problems during the COVID-19 pandemic, the most significant variable is sex; in this sense, in our study, 79.0% of participants with psychological distress were female. In addition, other variables that influence this vulnerability to psychological suffering are level of education (university studies: graduation, master’s, and doctorate) (75.8%), working outside the home (69.2%), conditions of the usual dwelling (where 59.2% lived in a flat), and living with children or young people under the age of 16 (50.2%). People who are unemployed (10.3%) are the ones with the lowest percentage of psychological distress. The results show that there are statistically significant differences between psychological problems and sex. There is no statistical relationship between psychological suffering and age, that is, age does not influence whether or not one is suffering. Other studies corroborate the results of our study regarding the increased risk for women of developing psychological distress throughout the COVID-19 pandemic, an aspect that can be interpreted as an individual and biological risk factor. On the other hand, due to the closing of schools during the pandemic, women suffered disproportionately from the burden of caregiving, with increased responsibilities of work and household chores [7,12,16].

Regarding the level of education as a predictor of psychological distress, our study shows that there is greater psychological distress in people with higher levels of education (university studies: graduation, master’s, and doctorate), with no evidence in other studies of the relationship between psychological suffering and education. The correlation presented by our study may indicate that the ease of access that people with a higher level of education have to credible scientific information and their perception of the severity of the virus based on scientific evidence—together with uncertainty about the direction the pandemic may take, as there is little knowledge about the virus, and means of treatment and control—can lead this group of people to develop fear and anxiety [31].

In relation to people living with children or young people under 16 years of age, other studies are in line with these results, being explained by the fear of contagion of the children, by the burden of the caregiver, and by the difficulty in providing playful and fun activities for children, creating a feeling of boredom and being still in time [7,12,28,32,33,34].

In our study, the unemployed sample showed the lowest psychological distress. On the other hand, it is those who have to work outside the home (69.2%) who have greater psychological suffering, which can be justified by the fear of contracting COVID-19 and transmitting it to others, inadequate protection against contamination, discrimination, overwork, and exhaustion. The results of the studies by Gomez-Salgado et al. [7] and Jeong et al. [33] are in line with our results. Data from a study conducted in China contradict our results and argue that psychological distress during the pandemic is related to unqualified and low-skilled jobs and unemployment, as these situations create distress related to the socio-economic situation [27].

The data presented in this study reveal that there is no statistical relationship between psychological distress and age. The evidence shows different results taking into account different age groups—that is, there are studies that show that the younger population is more likely to develop psychological suffering, because they have more difficulty in dealing with adversities and also in understanding that the pandemic is an extreme situation, which implies drastic changes in the lifestyle of a society and does not result from individual decisions [7,30]. Other studies reveal that psychological distress is greatest in people over 60 years of age, as they are part of the group most at risk of developing COVID-19 [32,35].

Regarding the presence of symptoms of COVID-19, the most frequent were headaches (46.6%), rhinorrhea (30.1%), myalgia (24.7%), cough (15.3%), sore throat (14.7%), and in a smaller percentages, diarrhea (9.7%), dizziness (6.9%), chills (5.2%), respiratory distress (3.6%), and fever above 38 °C for one day (1.1%). With the exception of respiratory distress and the presence of fever greater than 38 °C for one day, all other physical symptoms related to COVID-19 are a predisposing factor to the existence of psychological distress. Other studies have found myalgias, dizziness, chills, and odynophagia to be associated with greater psychological distress [17]. In a study conducted in Spain, the presence of headache, rhinorrhea, myalgias, cough, sore throat, diarrhea, dizziness, chills, difficulty breathing, and fever higher than 38 °C for one day are also associated with increased psychological distress [7]. 

Of the participants with COVID-19 symptoms (4.5%), 18.1% were tested—69.2% had a negative result, 22.2% had a positive result, and 8.7% did not know the result. Only 3.1% of respondents indicate that they were quarantined for having had contact with a person infected with COVID-19.

In our study, health-related variables (presence of chronic diseases; taking medication regularly; needing to attend consultations in a health centre, hospital, or clinic regularly; having recently performed COVID-19 tests; and self-assessment of health perception) were related to the presence of psychological distress, as had already been described in the studies by Shehata et al. [36], Cybulski et al. [37], and Ripoll et al. [38]. Regarding the evaluation of the perception of the health of our sample, the results show that the group of people who do not experience psychological distress expressed a better evaluation of their health compared to the group that had psychological distress, although both made a good self-assessment of their health. Other studies corroborate our results, describing a relationship between the existence of comorbidities and the presence of psychological distress in patients with COVID-19 and in the general population, but this relationship was not statistically significant [8,39].

Regarding contact history in the 14 days prior to this survey, of our sample, 36.1% of participants claimed to have had close contact with an individual who tested positive for COVID-19 [35]. One percent of individuals claimed to have had random contact, and 43% claimed to have maintained or not known if they had had contact with any person or material suspected of being infected with COVID-19. Regarding the presence of an infected person in the close circles of the participants, 91.2% stated that they did not have infected family members. All variables (close contact with an individual with confirmed infection, casual contact with an individual with confirmed infection, contact with any person or material suspected of being infected with COVID-19, any member of the infected family) relating to the contact history have a statistically significant relationship with the presence of psychological distress. Data from other studies show that a history of contact with an infected person or objects predisposes one to develop psychological distress, namely the presence of acute stress and post-traumatic stress, which arise from the feeling of danger and risk of contracting the infection [7,40,41].

In order to describe the limitations of the present study, it is worth mentioning that the cross-sectional design used does not allow establishing a cause-effect relationship, although it does provide a very valuable description of the impact of the COVID-19 pandemic on psychological distress at the specific moment of confinement and of the greatest escalation of the infection curve in Portugal. On the other hand, the sampling procedure was not random, and the study participants were collected through email lists to universities and professional associations. Moreover, the sex variable was not represented in the actual proportion of the Portuguese population. Furthermore, it was not possible to compare the results of the present study with those obtained during the development of other pandemics, since the measures adopted in the current situation differ from those implemented in those past situations. Similarly, the results obtained in Portugal cannot be compared with those obtained in studies conducted on the same topic in other countries. 

Future studies should carry out cause-effect analyses, perhaps at different epidemiological moments of the pandemic, to really study what has been and what will be the emotional impact of the COVID-19 pandemic in Portugal.

## 5. Conclusions

Having had close or casual contact with a person with confirmed or suspected COVID-19 infection, including family members, had a statistically significant relationship with the presence of psychological distress.

The predictor variables for psychological distress were sex, educational background, living with children or young people under 16 years of age, number of symptoms presented in the last 14 days, quarantine for presenting symptoms, having had contact with a person or material suspected of being infected by COVID-19, and having had close contact with a person with confirmed COVID-19 infection. The protective variables for psychological distress were being in telework and a good self-assessment of health status in the last 14 days.

These results show that there are factors that predispose the Portuguese population to develop psychological distress in the context of the COVID-19 pandemic, affecting their well-being and mental health. These should be considered when carrying out intervention programs suitable for pandemic situations, since they can design programs for the prevention of psychological distress in a pandemic context and psychological intervention programs when distress is already present, with the aim of promoting public health in general, and mental health in particular.

## Figures and Tables

**Table 1:** General Health Questionnaire (*n* = 2120).

Items	M (SD)
1. Have you been able to concentrate well on what you are doing?	2.40 (0.69)
2. Have your worries made you lose a lot of sleep?	2.47 (0.99)
3. Have you felt that you are playing a useful role in life?	1.98 (0.69)
4. Have you felt capable of making decisions?	2.09 (0.54)
5. Have you felt constantly overwhelmed and stressed?	2.68 (0.90)
6. Have you had the feeling that you cannot overcome your difficulties?	2.13 (0.88)
7. Have you been able to enjoy your normal daily activities?	2.62 (0.89)
8. Have you been able to adequately cope with problems?	2.26 (0.59)
9. Have you felt unhappy or depressed?	2.22 (0.99)
10. Have you lost confidence in yourself?	1.69 (0.85)
11. Have you thought that you are a worthless person?	1.44 (0.78)
12. Do you feel reasonably happy considering all the circumstances?	2.27 (0.67)
Scale total (over 12 points)	3.97 (3.43)
Presence of psychological distress (cut point ≥ 3)	(%)
Yes	57.2
No	42.8

**Table 2:** Association between sociodemographic variables and psychological distress during the COVID-19 pandemic (*n* = 2120).

		Psychological Distress				
	*n* (%)	No (*n* = 908)	Yes (*n* = 1212)	χ^2^/*t*	*p*	Effect Size
Sex						
Male	684 (32.3)	47.2	21.0	163.137	<0.001	0.277
Female	1436 (67.7)	52.8	79.0			
Age (mean (SD))	38.84 (13.00)	39.31 (14.60)	38.49 (11.66)	1.394	0.163	0.063
Marital Status						
Single	837 (39.5)	43.6	36.4	13.353	0.001	0.079
Married or living as a couple	1090 (51.4)	46.9	54.8			
Separated/divorced/widowed	193 (9.1)	9.5	8.8			
Educational level						
Secondary school	628 (29.6)	36.8	24.3	41.666	<0.001	0.140
University education (graduation)	721 (34.0)	28.9	37.9			
University education (master’s or PhD)	771 (36.4)	34.4	37.9			
Employment status						
Working away from home	1450 (68.4)	67.3	69.2	8.328	0.016	0.063
Working from home	474 (22.4)	24.9	20.5			
Not working	196 (9.2)	7.8	10.3			
Type of dwelling						
Flat or Apartment	1210 (57.1)	54.3	59.2	5.011	0.025	0.049
House	910 (42.9)	45.7	40.8			
Living with children or under-16 youngsters						
No	1146 (54.1)	59.8	49.8	21.108	<0.001	0.100
Yes	974 (45.9)	40.2	50.2			
Living with disabled people						
No	1994 (94.1)	94.6	93.6	0.850	0.357	0.020
Yes	126 (5.9)	6.4	5.4			

**Table 3:** Association between physical symptoms in the past 14 days and psychological distress during the COVID-19 pandemic (*n* = 2120).

		Psychological Distress				
	*n* (%)	No (*n* = 908)	Yes (*n* = 1212)	χ*^2^/t*	*p*	Effect Size
Fever (>38 °C for at least 1 day)						
No	2101 (99.1)	99.3	98.9	0.991	0.319	0.022
Yes	19 (0.9)	0.7	1.1			
Cough						
No	1848 (87.2)	90.5	84.7	16.021	<0.001	0.087
Yes	272 (12.8)	9.5	15.3			
Headache						
No	1350 (63.7)	77.4	53.4	129.709	<0.001	0.247
Yes	770 (36.3)	22.6	46.6			
Myalgia						
No	1702 (80.3)	86.9	75.3	43.855	<0.010	0.144
Yes	418 (19.7)	13.1	24.7			
Dizziness						
No	2013 (95.0)	97.5	93.1	20.948	<0.001	0.099
Yes	107 (5.0)	2.5	6.9			
Diarrhea						
No	1944 (91.7)	93.6	90.3	7.645	0.006	0.060
Yes	176 (8.3)	6.4	9.7			
Sore throat						
No	1870 (88.2)	92.1	85.3	22.785	<0.001	0.104
Yes	250 (11.8)	7.9	14.7			
Coryza						
No	1632 (77.0)	86.5	69.9	80.425	<0.001	0.195
Yes	488 (23.0)	13.5	30.1			
Chills						
No	2042 (96.3)	98.3	94.8	18.419	<0.001	0.093
Yes	78 (3.7)	1.7	5.2			
Breathing difficulty						
No	2055 (96.9)	97.7	96.4	3.032	0.082	0.038
Yes	65 (3.1)	2.3	3.6			
Number of symptoms (mean (SD))	1.24 (1.45)	0.80 (1.16)	1.58 (1.55)	−13.150	<0.001	0.559

**Table 4:** Association between health-related variables and psychological distress during the COVID-19 pandemic (*n* = 2120).

		Psychological Distress				
	*n* (%)	No (*n* = 908)	Yes (*n* = 1212)	χ^2^/*t*	*p*	Effect Size
Chronic diseases						
No	1505 (71.0)	75.4	67.7	15.058	<0.001	0.084
Yes	614 (29.0)	24.6	32.3			
Currently taking any medication						
No	1361 (64.2)	70.9	59.2	31.270	<0.001	0.121
Yes	759 (35.8)	29.1	40.8			
Health care in a health centre, clinic, or hospital in the past 14 days						
No	2007 (94.7)	96.0	93.6	5.868	0.015	0.053
Yes	113 (5.3)	4	6.4			
Recent hospitalization in the past 14 days						
No	2111 (99.6)	99.8	99.4	1.568	0.211	0.027
Yes	9 (0.4)	0.2	0.6			
Self-rated health in the past 14 days *	4.02 (0.81)	4.26 (0.73)	3.85 (0.83)	11.930	<0.001	0.520
Recent testing for COVID-19 in the past 14 days						
No	1736 (81.9)	86.6	78.4	23.424	<0.001	0.105
Yes	384 (18.1)	13.4	21.6			
Recent quarantine in the past 14 days for having symptoms (*n* = 2106) **		*n* = 2040	*n* = 66			
No	2040 (96.9)	98.7	95.5	16.725	<0.001	0.089
Yes	66 (3.1)	1.3	4.5			

Note: * Likert-type scale from 1 (very bad) to 5 (very good); ** Fisher’s exact test.

**Table 5:** Association between contact history variables in the past 14 days and psychological distress during the COVID-19 pandemic (*n* = 2120).

		Psychological Distress				
	*n* (%)	No (*n* = 908)	Yes (*n* = 1212)	χ^2^	*p*	Effect Size
Close contact with an individual with confirmed infection with COVID-19						
No	1354 (63.9)	73.0	57.0	57.618	<0.001	0.165
Yes or does not know	766 (36.1)	27.0	43.0			
Casual contact with an individual with confirmed infection with COVID-19						
No	1333 (62.9)	70.7	57.0	41.690	<0.001	0.140
Yes or does not know	787 (37.1)	29.3	43.0			
Contact with any person or material suspicious of being infected with COVID-19						
No	1208 (57.0)	66.5	49.8	58.953	<0.001	0.167
Yes or does not know	912 (43.0)	33.5	50.2			
Any infected family member						
No	1933 (91.2)	93.6	89.4	11.691	0.001	0.074
Yes or does not know	187 (8.8)	6.4	10.6			

**Table 6:** Prediction of psychological distress.

	Variables	Model 1 OR (95% CI) Sociodemographic	Model 2 OR (95% CI) Physical Symptoms	Model 3 OR (95% CI) Health Related	Model 4 OR (95% CI) Contact History	Model 5 Global Model OR (95% CI)
	Regression Model	R^2^ = 0.127 (79.3/45.8%)	R^2^ = 0.160 (81.3/44.3%)	R^2^ = 0.168 (83.8/42.8%)	R^2^ = 0.131 (80/45.6%)	R^2^ = 0.226 (78/54.9%)
Sociodemographic	Sex (ref. males)	3.142 ** (2.566, 3.848)	2.744 ** (2.254, 3.340)	2.898 ** (2.373, 3.539)	3.172 ** (2.616, 3.845)	2.448 ** (1.980, 3.028)
	Marital Status (ref. single)					
	Married or living as a couple	0.938 (0.755, 1.167)	NA	NA	NA	NA
	Separated/divorced/widowed	0.763 (0.542, 1.075)	NA	NA	NA	NA
	Educational level (ref. secondary school)					
	University education graduation)	1.541 ** (1.210, 1.962)	NA	NA	NA	1.419 ** (1.170, 1.820)
	University education (master’s or PhD)	1.327 * (1.039, 1.695)	NA	NA	NA	1.163 (0.906, 1.494)
	Employment status (ref. working away home)					
	Working from home	0.679 ** (0.541, 0.852)	NA	NA	NA	0.784 * (0.618, 0.995)
	Not working	1.091 (0.784, 1.520)	NA	NA	NA	0.961 (0.684, 1.351)
	Type of dwelling (ref. apartment)	0.856 (0.712, 1.030)	NA	NA	NA	NA
	Living with children or under-16 youngsters (ref. no)	1.587 ** (1.309, 1.926)	NA	NA	NA	1.580 ** (1.304, 1.915)
Physical symptoms	Number of symptoms	NA	1.444 ** (1.339, 1.558)	NA	NA	1.327 ** (1.224, 1.440)
Health related	Chronic diseases (ref. no)	NA	NA	0.898 (0.695, 1.160)	NA	NA
	Currently taking any medication (ref. no)	NA	NA	1.184 (0.929, 1.509)	NA	NA
	Health care in a health centre, clinic, or hospital in the past 14 days (ref. no)	NA	NA	1.017 (0.653, 1.582)	NA	NA
	Self-rated health in the past 14 days*	NA	NA	0.563 ** (0.496, 0.640)	NA	0.672 ** (0.589, 0.767)
	Recent testing for COVID-19 in the past 14 days (ref. no)	NA	NA	1.230 (0.953, 1.588)	NA	NA
	Recent quarantine in the past 14 days for having symptoms (ref. no)	NA	NA	2.878 ** (1.456, 5.687)	NA	2.443 * (1.214, 4.913)
Contact history	Close contact with an individual with confirmed infection with COVID-19 (ref. no)	NA	NA	NA	1.499 ** (1.120, 2.007)	1.347 * (1.031, 1.759)
	Casual contact with an individual with confirmed infection with COVID-19 (ref. no)	NA	NA	NA	0.948 (0.707, 1.271)	NA
	Contact with any person or material suspected of being infected with COVID-19 (ref. no)	NA	NA	NA	1.437 ** (1.108, 1.863)	1.237 (0.958, 1.598)
	Any infected family member (ref. no)	NA	NA	NA	1.304 (0.925, 1.836)	NA

* *p* < 0.05; ** *p* < 0.01; NA: not applicable; R^2^ = model explained variance (sensitivity/specificity); OR (95% CI): odds ratio (confidence interval at the 95% level).

## Data Availability

All data are available within this article.
